# Sialic Acid and Sialylated Oligosaccharide Supplementation during Lactation Improves Learning and Memory in Rats

**DOI:** 10.3390/nu10101519

**Published:** 2018-10-16

**Authors:** Elena Oliveros, Enrique Vázquez, Alejandro Barranco, María Ramírez, Agnes Gruart, Jose María Delgado-García, Rachael Buck, Ricardo Rueda, María J. Martín

**Affiliations:** 1 R&D Abbott Nutrition, 18004 Granada, Spain; enrique.vazquez@abbott.com (E.V.); alejandro.barranco@abbott.com (A.B.); maria.ramirez@abbott.com (M.R.); ricardo.rueda@abbott.com (R.R.); maria.j.martin@abbott.com (M.J.M.); 2 Doctoral programme in Biomedicine, School of Health Sciences, University of Granada, 18071 Granada, Spain; 3 Division of Neurosciences, Pablo de Olavide University, 41013 Seville, Spain; agrumas@upo.es (A.G.); jmdelgar@upo.es (J.M.D.-G.); 4 R&D Abbott Nutrition, Columbus, OH 43219, USA; rachael.buck@abbott.com

**Keywords:** milk oligosaccharides, infant formula, 6′-sialyllactose, cognitive development, sialic acid

## Abstract

Sialic acids (Sia) are postulated to improve cognitive abilities. This study evaluated Sia effects on rat behavior when administered in a free form as N-acetylneuraminic acid (Neu5Ac) or conjugated as 6′-sialyllactose (6′-SL). Rat milk contains Sia, which peaks at Postnatal Day 9 and drops to a minimum by Day 15. To bypass this Sia peak, a cohort of foster mothers was used to raise the experimental pups. A group of pups received a daily oral supplementation of Neu5Ac to mimic the amount naturally present in rat milk, and another group received the same molar amount of Sia as 6′-SL. The control group received water. After weaning, rats were submitted to behavioral evaluation. One year later, behavior was re-evaluated, and in vivo long-term potentiation (LTP) was performed. Brain samples were collected and analyzed at both ages. Adult rats who received Sia performed significantly better in the behavioral assessment and showed an enhanced LTP compared to controls. Within Sia groups, 6′-SL rats showed better scores in some cognitive outcomes compared to Neu5Ac rats. At weaning, an effect on polysialylated-neural cell adhesion molecule (PSA-NCAM) levels in the frontal cortex was only observed in 6′-SL fed rats. Providing Sia during lactation, especially as 6′-SL, improves memory and LTP in adult rats.

## 1. Introduction

Breast feeding is associated with multiple health benefits. Infants fed human milk have lower rates of intestinal and autoimmune diseases and food allergies and show higher intellectual quotient (IQ) scores compared to formula-fed infants [1]. The unique composition of human milk plays a key role in optimal development of newborns, and sialic acids (Sia) are considered as one of the components responsible for the multiple benefits. They are present in most mammalian milks, mainly conjugated to proteins and oligosaccharides, but also as free monosaccharides. Within this last group of monosaccharides, there are some key core structures such as N-acetylneuraminic acid (Neu5Ac), N-glycolylneuraminic acid (Neu5Gc) and deaminated neuraminic acid (Kdn), which encompass the so-called Sia molecules [2]. Neu5Ac is the only form of Sia synthesized by humans [2] and is present throughout the body, including brain [3].

Most of the Sia in human milk, approximately 73% [4], are bound to oligosaccharides, generating sialyloligosaccharides, which represent a significant fraction of human milk oligosaccharides (HMOs). Oligosaccharides in human milk are at a higher concentration than in other mammalian milks, ranging from 13.3–23 g/L in colostrum to 3.5–14 g/L in mature milk [4,5], with structural diversity and over 200 different molecules identified so far [6]. Conversely, bovine milk, which is the basis of most infant formula, has an oligosaccharide content over 100-fold [7] lower than human milk. Although goat milk-based infant formula is now available, its use is not as extensive as bovine milk-based infant formulas. Interestingly, goat milk oligosaccharides have significant structural similarities to human milk oligosaccharides [8].

Milk oligosaccharides from any mammal consist of acidic oligosaccharides, predominantly sialylated ones, and neutral oligosaccharides, predominantly fucosylated compounds. There is a significant difference in the distribution of these compounds between human and bovine milk [9]. Sialylated structures represent 70% of bovine milk oligosaccharides compared with 10–20% in human milk [8], while fucosylated glycans are the most abundant form in human milk [10]. Within sialylated compounds, a recent study showed 6′-sialyllactose (6′-SL) is the major representative during the first two months, when it starts to sharply decrease so that 3′-sialyllactose becomes the predominant sialyl-oligosaccharide of human milk beyond the fourth month [11].

Milk oligosaccharides have been linked to different biological functions in the newborn, such as anti-infective activity [12,13], immune system and gastrointestinal development [14,15], modulation of inflammation [16], bifidogenic activity [17], modulation of gut motility and activation of enteric neurons [18] and even enhancement of central nervous system (CNS) functions [19]. Sia has been described as an essential nutrient in the development of infant brain [20], since its highest concentration in milk during early lactation concurs with a rapid increase of brain gangliosides [21]. Furthermore, the content of gangliosides and protein-bound sialic acid is higher in the brain of breast-fed infants in comparison to formula-fed infants [22]. Sia is a key component of brain gangliosides and polysialic acid (polySia) attached to the oligosaccharide chains on neural cell adhesion molecule (NCAM); both brain gangliosides and polysialylated-NCAM (PSA-NCAM) are crucial to ensure synaptic connections and memory formation or neuronal outgrowth [3,20].

HMOs and, by extension, sialyloligosaccharides have been reported as important factors for optimal development and maturation of the immune system [23]. HMOs are also prebiotics favoring the growth of healthy gut microbiota and as pathogen decoys, inhibiting their adhesion to the intestinal mucosa [24,25,26].

Several studies performed both in rodents [27] and pigs [28] have shown a relationship between Sia and cognitive function. The mechanism of action for the role of Sia on cognitive development has not been proven to date. However, recent research has shown the effects of HMOs on the gut-brain axis (GBA) in rodents [29,30], which compliments their role as prebiotics. The gut-brain axis consists of a bidirectional interaction between the central and the enteric nervous system [31]. The influences of gut microbiota on human health and disease is an important topic in medicine that is believed to occur via gut-brain microbe communication [32]. A recent study demonstrated associations between gut microbiota and cognition in infants [33].

The first two years of life are a critical window for brain development, not only for rapid proliferation and growth of neural cells, but also for increases in synapse connections [34]. Thus, adequate early life nutrition is crucial to ensure optimal cognitive development in infants and children.

The present study was aimed at determining if early Sia supplementation, as a free or conjugated form, plays a role during neural development in rats and whether effects could be detected later in life. For that purpose, rat pups were orally supplemented with Neu5Ac or 6′-SL during the lactation period and were evaluated using behavioral tests and electrophysiological measurements in both young and adult rats.

## 2. Materials and Methods

### 2.1. Animals

Pregnant Sprague-Dawley rats (*n* = 47) in the second week of gestation were purchased from Charles River (Charles River, France) and kept under controlled environmental conditions of temperature (22 °C ± 2), humidity (55% ± 10) and lighting (12 h light/dark cycles). Rats were fed a standard diet ad libitum. Animal experimental protocols were approved by the Ethics Committee of the Estación Experimental del Zaidín-CSIC (Consejo Superior de Investigaciones Científicas, Granada, Spain. Approval number: CBA EEZ-2011/20), and the experiment was performed in accordance with the Spanish and European regulations for the care and use of experimental animals for research.

### 2.2. Experimental Design

**Scheme 1:**
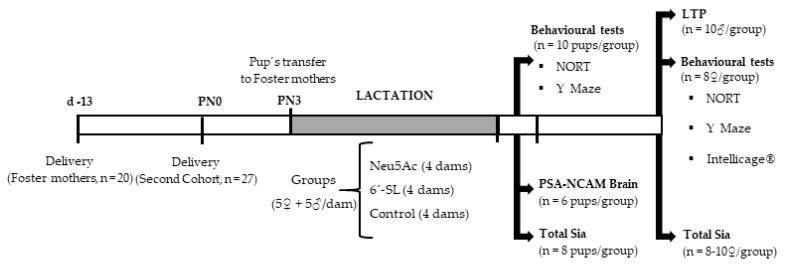
Experimental design scheme. PN, postnatal day; Neu5Ac, N-acetylneuraminic acid; 6′-SL, 6′-sialyllactose; NORT, Novel Object Recognition Test; PSA-NCAM, polysialylated-neural cell adhesion molecule; LTP, long-term potentiation; Sia, sialic acids; ♀, female; ♂, male.

Rodent milk contains sialylated oligosaccharides, in particular 3′-sialyllactose (3′-SL) and 6′-SL [35]. In rat milk, total Sia levels peak at Postnatal Day 9 (PN9), sharply drop until Day 15 and then slightly decrease until the end of lactation [36]. To bypass this peak of Sia, two cohorts of pregnant rats were used with a time lag on the delivery day of 13 days, as shown in the experimental design scheme (see Scheme 1). Animals were maintained on a chow diet for gestation, lactation and growth (2018 Teklad Global 18% Protein Diet) until delivery. The first cohort (*n* = 20), hereafter called foster mothers, was kept with its respective offspring until PN16. Experimental pups were born from the second cohort of mothers (*n* = 27) and remained with them until PN3. On that day, 3-day-old pups were removed from their dams, sexed and randomly distributed into 10 pups (5 males and 5 females) groups with balanced weights and litters. Subsequently, they substituted for the 16 day-old pups in foster mother cages. These dams willingly accepted the new litters and took care of them for the rest of the lactation period. Afterwards, foster mothers and their litters were then distributed into 3 experimental groups of 4 dams each.

Since the content of sialic acid in rat milk reaches the lowest values from PN16, all pups raised by foster mothers received less Sia from milk than normally raised rats during early development. To compensate for the deficiency of Sia in milk, one group of pups received an oral supplementation of Neu5Ac from PN3 to weaning, and another group received the same molar amount of Sia given as 6′-SL. The control pups were given water and, therefore, received approximately 5-times less Sia than normally raised rats. In contrast, the 2 experimental groups received the same Sia levels naturally present in rat milk, but from two different sources. Therefore, the model assessed adequate Sia intake during lactation and the influence of the form in which it was provided.

Pups remained undisturbed with the foster rats except for a brief time while they received supplementation (8 times/day; max. 5 min/time). After weaning, all mothers and 2 pups per litter were sacrificed by an intraperitoneal overdose of anesthetic. Brains obtained from the pups were separated into hemispheres. One hemisphere was analyzed to determine Sia content at weaning by high performance liquid chromatography (HPLC), and the other one was used to determine NCAM and PSA-NCAM expression in the frontal cortex by Western blotting. Another set of weaned rats was submitted to classical behavioral tests such as NORT (Novel Object Recognition Test) and Y maze tests. Pups were then maintained for 1 year on a standard chow diet. At that age, males were used for LTP measurements; meanwhile, females were subjected again to psychological tests to evaluate long-term effects on learning and memory. Rats were then sacrificed, and brains were analyzed by HPLC to determine Sia content at adulthood.

### 2.3. Sialic Acid Doses

Rat milk contains both forms of free Sia, Neu5Ac and Neu5Gc; since Neu5Ac is the most abundant in humans [3] and is present throughout the body including brain, this was the form chosen as a free Sia source to be used in the supplementation.

**Figure 1:**
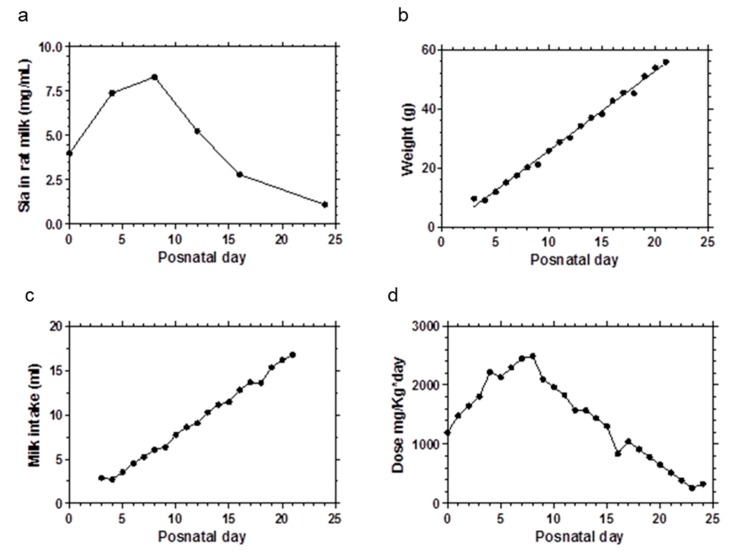
(**a**) Sia concentration (mg/mL) in rat milk throughout the lactation period per the literature [36]. (**b**) Experimental data of rat pups’ weight evolution from birth until weaning. (**c**) Estimated data of milk volume intake (mL) in rat pups from birth until weaning per the literature [37]. (**d**) Sialic acid dose (mg/kg/day) that rat pups were receiving from rat milk considering the data shown in graphs (**a**–**c**).

Rat milk concentration of sialic acid varies through the lactation period having a Sia peak (≈8 mg/mL) at PN9 and dropping to lower levels by Postnatal Day 15 [35] (Figure 1a).

To determine the effects of Sia, a model was designed in which one of the groups received less Sia than that from normal lactation, while the other groups received the same levels of Sia as naturally-nursed pups, either in the free form Neu5Ac or as 6′-SL. The daily dose of Sia was calculated based on Sia content of rat milk, pup body weights and milk intake during lactation according to data found in the literature [36,37] and to internal data from previous experiments (Figure 1b). Figure 1d shows the theoretical Sia dose (mg/kg weight/day) that rat pups would normally receive from rat milk. Equimolar stock solutions with Neu5Ac and 6′-SL were prepared. Variable volumes of these stock solutions were given to the pups per day to adjust the Sia dose, as shown in Figure 1d.

### 2.4. Analytical Determination of Sialic Acid Content by HPLC

Right hemispheres were analyzed to determine the total amount of sialic acid. After homogenization of the brain, 35 mg were weighed and re-suspended at 0.2 mg/µL in deionized water. Sia were released by mild hydrolysis in 0.2N H_2_SO_4_ at 80 °C for 1 h. After filtering and centrifuging during 30 min at 10,000 rpm, supernatants were used for derivatization with 5-(difluoromethoxy)-2-mercapto-1H-benzimidazole (DMB) as described by Hara et al. [38] with some modifications. Thus, 50 μL of samples and 50 μL of DMB reagent (8 mM DMB, 1.5 M acetic acid, 14 mM sodium hydrosulfite, 0.8 M 2-mercaptoethanol (Sigma Aldrich, Saint Louis, MO, USA)) were kept for 2.5 h at 50 °C in the dark. A volume of 10 μL of the derivatized solution was injected on an Alliance 2695 HPLC system equipped with a 474-fluorescence detector from Waters (Mildford, MA, USA) as described by Martin et al. [39]. A LiChrosorb RP-18 column (5 μm, 250 mm × 4.6 mm) with a LiChrosorb RP-18 guard cartridge (5 μm), both from Supelco (Bellefonte, PA, USA), was used. DMB derivatives of Sia were isocratically eluted using 7% (*v/v*) methanol, 8% (*v/v*) acetonitrile in water for 40 min at a flow rate of 0.9 mL min^−1^. All injections were performed at room temperature. The eluent was monitored for fluorescence at 373 nm (excitation wavelength) and 448 nm (emission wavelength). The gain was fixed at 1, and the attenuation at 64 for the 474-fluorescence detector. A set of standards (25–250 ng Neu5Ac) was injected with every set of samples. Data were integrated by the Millennium 4.0 software (Waters, Milford, MA, USA) coupled to the HPLC system. Standard regression and sample quantitation were calculated by Microsoft Excel 2011 and Graph Pad (Prism 4, San Diego, CA, USA).

### 2.5. Western Blotting

For Western analysis, ≈15–20 mg of frontal cortex samples of pups at weaning (PN22) were homogenized in cold suspension buffer (PBS, 0.1% TritonX100 and protease inhibitor cocktail from Sigma Aldrich, Saint Louis, MO, USA), centrifuged at 10,000 *g* for 15 min, and the supernatant collected. The protein content was determined using the Bradford assay (Bio-Rad, Hercules, CA, USA). Ten micrograms of protein, diluted in phosphate-buffered saline (PBS), were loaded into Criterion XT 4–20% Bis-Tris gels (Bio-Rad). Separation was carried out using MOPS buffer (0.05 M 3-Morpholinopropane-1-sulfonic acid (MOPS), 0.05 M Tris base, 0.003 M SDS, 0.8 mM EDTA) and run at 200 V for 45 min. Separated proteins were transferred onto nitrocellulose membranes for 3.5 h using a Trans-Blot electrophoresis transfer cell. Six samples were run per group. After blocking non-specific binding sites for 1 h with 3% bovine serum albumin (BSA, Sigma Aldrich, Saint Louis, MO, USA) in TBS-TritonX100 (0.025% TritonX100, 20 mM Tris base, 150 mM NaCl, pH 7.6), blots were incubated overnight at 4 °C with one of the following monoclonal antibodies: anti-NCAM (Santa Cruz, CA, USA) at 1:2500; anti-PSA-NCAM (Millipore/Merck, Darmstadt, Germany) at 1:1000. All the antibodies were diluted in TBS containing 1% BSA. After three quick washes in TBS-TritonX100, NCAM blots were incubated for 2 h at room temperature with anti-IgG HRP at 1:5000 (Sigma Aldrich), while PSA-NCAM were incubated with anti-IgG HRP (Bethyl laboratories, Montgomery, TX, USA) at 1:5000 for 2 h. β-actin was used as a control for quantitation and detected with monoclonal antibody (Sigma Aldrich, 1:5000). After five washes in TBS-TritonX100, membranes were developed using Pierce Supersignal West-Pico substrate (Thermo Fisher Scientific, Waltham, MA, USA) and quantified using a Chemidoc XRS system from Bio-Rad (Hercules, CA, USA).

### 2.6. Long-Term Potentiation Measurement

One-year old male offspring were assigned to this analysis. Animals were anesthetized and implanted with stimulating and recording electrodes in the hippocampus to measure LTP in vivo, as previously described [40]. Based on prior research [41,42], animals were implanted with stimulating electrodes at the Schaffer collateral-commissural pathway of the dorsal hippocampus (3.5 mm lateral and 3.2 mm posterior to Bregma) as per stereotaxic coordinates. Four recording electrodes were also implanted targeting the ipsilateral stratum radiatum beneath the CA1 area (2.5 mm lateral and 3.6 mm posterior to Bregma). Stimulation occurred, and the field excitatory post-synaptic potential (fEPSP) was recorded. After the high-frequency stimulation (HFS) protocols, fEPSPs were recorded for 30 min. Additional 15-min recording sessions were carried out during the following days.

### 2.7. Classical Behavioral Tests

A video tracking system, Sony Camera SSC-G213A (Sony Electronics Inc., Park Ridge, NJ, USA), was used to record all animal trials. Rat performances were then analyzed offline using the analytical software Viewer (Biobserve GmbH, Bonn, Germany) by a technician that was blinded to treatment. An alcohol dilution was used to clean the surface of Y maze and NORT structures after each animal performance to prevent odor cues.

#### 2.7.1. Novel Object Recognition Test

NORT is based on the natural tendency of rodents to explore novel stimuli presented to them; thus, when novel and familiar stimuli are present at the same time, the novel one will be naturally explored for longer [43]. Therefore, this test was run in a black opened plastic chamber (40 × 40 cm), known as the arena. Three objects differing in material, form and color were chosen. After 3 days of habituation to the arena, an acquisition phase took place, and the animals were faced with two different objects (familiar objects) for 10 min. One day later, animals were submitted to a retention test in which a new object (novel object) substituted one of the familiar objects, allowing the animals to explore them for 5 min. Animals were considered as exploring the objects when they approached their whiskers at approximately 1–2 cm or licked them; exploration was never considered when they sat on the objects [44]. The key parameter evaluated in this task was time spent by the rats exploring each object, novel or familiar, during the retention phase.

#### 2.7.2. Y Maze with Blocked Arm Test

The Y maze with blocked arm test measures the ability of rodents to explore new environments and assesses exploratory behavior and memory [45]. The experimental design used was that proposed by Dellu et al. [46] with some modifications. A Y-shape stainless-steeled structure (20 × 10.5 × 50 cm) was used. There are two consecutive phases in the paradigm: an acquisition trial and a retention test. During the acquisition trial, one of the three arms of the maze was blocked (novel arm), and rats could explore the other two arms for 15 min. Four hours later, animals went through the test session in which all arms were open to be explored for 5 min. Novelty was represented by the arm that was not accessible in the acquisition phase. When introduced in the maze after a few hours, animal normal behavior should be to explore first the previously hidden arm; thus, to evaluate animal memory skills, it was analyzed whether animals went first into the novel arm.

### 2.8. IntelliCage^®^ Protocol

The IntelliCage^®^ (NewBehavior AG, Zurich, Switzerland) is a computer-based, fully-automated testing apparatus used to analyze the spontaneous and learning behavior of rodents. This system consists of a cage that presents 4 operant conditioning corners, which can locate one rat at a time. Each corner is equipped with 2 motorized doors, which block or allow access to water bottles placed on both sides of the corner. When a rat tries to access through whichever of the 2 doors (nose poke action), the interruption of a light-beam sensor at either door triggers one or the other doors to open and allow access to a water bottle. Radio frequency identification (RFID) transponders are implanted under the rat skin, allowing for individual recognition; thus, rat entries into the corners are detected through RFID antennas located there. Using this technology, conditioning protocols can be implemented to evaluate the behavioral activities of the rats. Animals were implanted with the RFID-transponders, and 1 day later, they were placed into the IntelliCage^®^ (*n* = 8 rats/group) and maintained there during 2 weeks for habituation and testing. The habituation process was counted with different stages: 1 day of free exploration with all doors opened; 5 days in which doors were opened only upon a visit to the corner; in the following 2 days, doors opened after a nose poke in the right place; in the last 4 days, one nose poke opened any door only during two drinking sessions of 90 min per night, while the doors remained closed during the rest of the night and day.

Thereafter, a paradigm to measure cognitive outcomes was tested by restricting water access to a specific corner and time periods. During the two-day test, access to water was restricted to one corner (correct corner) only during the two drinking sessions. If the rat nose poked any other corner, water was not available. Each rat was randomly assigned a correct corner. Every visit to this assigned corner was counted as correct, while incorrect visits were those to any other corner. Thus, only rats with adequate cognitive skills could learn which is the correct corner in this place learning paradigm.

### 2.9. Statistical Analysis

Data are presented as the mean ± SEM. Graph displays and statistical analysis were done using SPSS (SPSS Inc., Chicago, IL, USA) and GraphPad (GraphPad Inc., La Jolla, CA, USA) software. Comparisons between groups were performed by Student’s *t*-test and one-way analysis of variance (ANOVA) in NORT and Western blotting. Statistical differences between groups were determined with a two-way repeated measure of the analysis of variance (ANOVA) in LTP measurements. The Y maze test and IntelliCage^®^ paradigm were analyzed through a contingency table analysis using Fisher’s test. The significance level was established at *p* < 0.05 for all tests.

## 3. Results

### 3.1. Sialic Acid Content in Brain

The content of Neu5Ac in the right brain hemisphere of rats at weaning (PN22) and at 1 year of age is shown in Table 1. Brain concentration of sialic acid (µg NeuAc/mg brain) did not show significant differences among groups at any age.

### 3.2. Western Blot

Frontal cortex samples of weaned pups (PN22) were analyzed to determine NCAM and PSA-NCAM expression by Western blotting (6 pups/group). Actin was used as a control for quantitation. The control group was used as the reference group and ratios were calculated between normalized values and the density mean for the control group.

**Figure 2:**
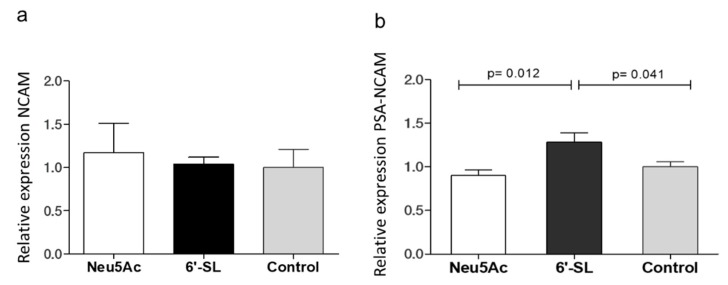
Relative protein expression of NCAM (**a**) and PSA-NCAM (**b**) in frontal cortex samples of rat pups of the three experimental groups at Postnatal Day 22. One-way ANOVA and Student’s *t*-test were used for statistical analysis. Data are expressed as the mean ± SEM (*n* = 6 rats/group), *p* < 0.05 denotes significant differences. NCAM, neural cell adhesion molecule; PSA-NCAM, polysialylated-neural cell adhesion molecule; Neu5Ac, N-acetylneuraminic acid; 6′-SL, 6′-sialyllactose.

As shown in Figure 2, no differences among groups related to NCAM expression (*p* = 0.8819) were found. However, rats fed 6′-SL in the lactation period expressed significantly more PSA-NCAM in the frontal cortex when compared to rats supplemented with Neu5Ac (*p* = 0.012) or control animals (*p* = 0.041).

### 3.3. In Vivo LTP

**Figure 3:**
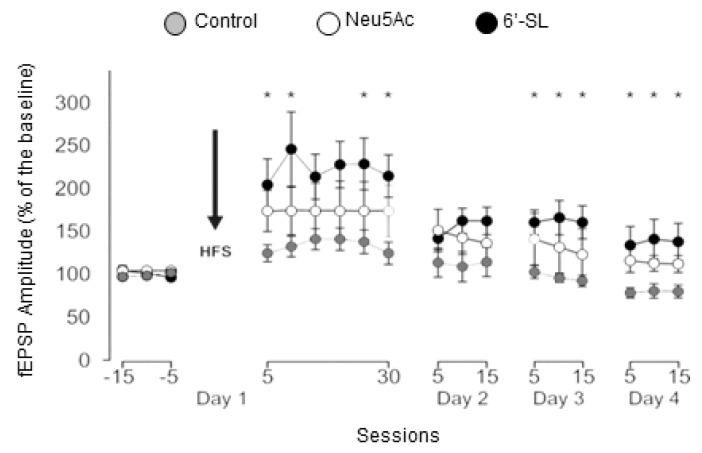
Experimentally-evoked LTP in the groups of animals at one year of age. Data collected from the three groups of animals before and after high-frequency stimulation (HFS) session (indicated by the downward arrow). The HFS was presented after 15 min of baseline recordings. LTP evolution was followed for up to four days. Statistical differences between groups were determined with a two-way repeated measure of the analysis of variance (ANOVA). The three groups of animals presented a significant (*p* ≤ 0.05) LTP in relation with baseline values. In addition, field excitatory post-synaptic potential (fEPSP) values evoked after the HFS session were significantly larger (*, *p* ≤ 0.05) for the 6′-SL group in comparison with values collected from controls. Data are represented as the mean ± SEM (*n* = 10 animals/group). Neu5Ac, N-acetylneuraminic acid; 6′-SL, 6′-sialyllactose.

At one-year of age, male rats were submitted to in vivo LTP (10 rats/group). A significant response in LTP was evoked in all the groups following the HFS session (Figure 3). LTP responses were significantly improved in male rats supplemented with 6′-SL during lactation compared to controls. Differences were detectable several days after the stimulation. Neu5Ac group also reached a more intense LTP than the control group, although the difference was not statistically significant.

### 3.4. Classical Behavioral Tests

A behavioral assessment with different paradigms such as NORT and the Y maze test was performed at weaning and when animals were one year old. Results obtained at weaning (*n* = 10 pups/group) did not show any difference in performance of the three groups tested (data not shown). Conversely, data found at adult age (*n* = 8 rats/group) were more conclusive.

#### 3.4.1. NORT

**Figure 4:**
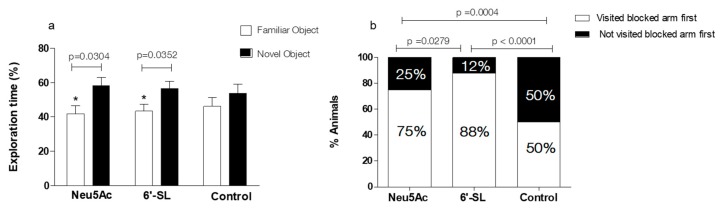
(**a**) Percentage of time spent by the animals exploring a familiar object against a novel object in NORT at one year of age. One-way ANOVA and Student’s *t*-test were applied for statistical analysis; *, *p* < 0.05 denotes significant differences. (**b**) Percentage of animals that chose the novel arm first in the Y maze during the retention test versus those that did not. Fisher’s test was applied for statistical analysis; *p* < 0.05 denotes significant differences (*n* = 8 rats/group). Neu5Ac, N-acetylneuraminic acid; 6′-SL, 6′-sialyllactose.

Regarding NORT, the key parameter to study was the time spent exploring the novel object versus the familiar one in the retention phase. Thus, animals with good cognitive abilities tended to explore the novel object for longer. As shown in Figure 4a, rats belonging to the groups that received Sia supplementation, both Neu5Ac and 6′-SL, spent a significantly longer time exploring the novel object than the familiar one. A significant difference in the exploration time of both objects was not found in the control group.

#### 3.4.2. Y Maze with Blocked Arm Test

As for the Y maze task, one-year old rats that received Neu5Ac or 6′-SL during lactation performed significantly better than the control group by clearly identifying the blocked arm. During the retention test, rats with superior cognitive abilities explored the arm first, since it represents novelty. Figure 4b shows the percentage of animals from each group that visited the previously blocked arm first versus the percentage of animals that chose one of the other two arms as their first option. Rats receiving Sia achieved the best score, i.e., 88% of 6′-SL animals visited the novel arm first and 75% of Neu5Ac rats. By contrast, the control group visited the novel arm with the same probability as the other two combined. From a statistical standpoint, the 6′-SL group score was significantly higher, not only when compared to the control group (*p* < 0.0001), but also compared to the Neu5Ac group (*p* = 0.0279). There were also significant differences between Neu5Ac rats and control animals (*p* = 0.0004).

### 3.5. IntelliCage^®^ Protocol

**Figure 5:**
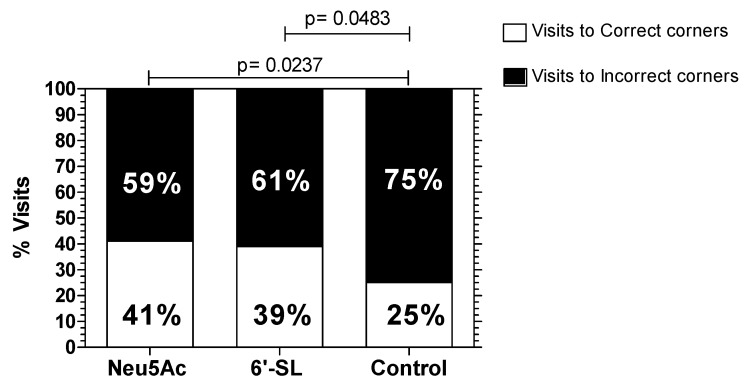
Percentage of total visits to the correct corner versus incorrect corner in the IntelliCage^®^ paradigm for each group of animals during the four drinking sessions of the two days of place learning. Fisher’s test was applied for statistical analysis; *p* < 0.05 denotes significant differences; *n* = 8 rats/group; one year of age. Neu5Ac, N-acetylneuraminic acid; 6′-SL, 6′-sialyllactose.

In the place learning paradigm performed with the IntelliCage^®^ system (*n* = 8 rats/group), results were aligned to the behavior observed in the classical tests. As previously explained, access to water was restricted to the “correct corner” during the two drinking sessions of two consecutive nights. The percentages of visits to the correct corner and visits to incorrect corners were analyzed. Neu5Ac and 6′-SL groups obtained 41% and 39% correct visits, respectively, while the control group reached 25% of visits to the correct corner (Figure 5). Thereby, groups supplemented with Neu5Ac or 6′-SL exhibited a significantly better performance compared to the control group.

## 4. Discussion

In the current study, the impact of two forms of sialic acid, either free form or conjugated given during lactation, on cognitive skills later in life was assessed. We evaluated cognitive functions with behavioral and electrophysiological measurements, demonstrating that these sources of Sia given at early stages after birth maintain cognitive function in adulthood when compared to a group that received a lower amount of Sia, and that the provision of Sia as 6′-SL may confer some advantages over the use of the free form.

Pioneering studies in the 1980s showed that exogenous Sia could be incorporated into brain gangliosides and glycoproteins when injected intraperitoneally in rat pups [27]. Wang et al. investigated the metabolic fate of intravenously-administrated ^14^C-Neu5Ac in piglets, concluding that an exogenous source of sialic acid could cross the blood-brain barrier and be incorporated into various tissues [47]. Carlson and House also demonstrated that Sia given by intraperitoneal injection or orally significantly impacted the concentration of brain gangliosides and glycoproteins [48]. Another study confirmed the impact of orally-administered sialic acid on cortical ganglioside concentration after feeding rat pups a solid diet prepared with different Sia doses from PN17–31. Cortical gangliosides Sia concentration was significantly higher in rats that received a Sia-supplemented diet provided by a protein-bound source of Sia (casein glycomacropeptide) [49].

Considerable research has been carried out to date to elucidate the effects of supplementation with Sia early in life on brain composition and behavior. In a study by Morgan and Winick [27], rat pups were intraperitoneally injected with Neu5Ac or glucose (control group), and an improvement of performance was observed in the Neu5Ac group. A piglet model was used by Wang et al. [28] to evaluate the effect of a sow milk replacer supplemented with several doses of casein glycomacropeptide for 35 days after weaning. Neu5Ac brain concentration, expression of two learning associated genes and learning and memory abilities were positively impacted by the Sia supplementation. However, a research team has recently reported two studies in which supplementation with sialyllactose from PN2–PN22 or PN32 in piglets showed no significant effect on recognition memory [50], but did show a sialic acid increase in hippocampus, prefrontal cortex and corpus callosum [51].

The novelty of the present work is three-fold: (1) the use of a unique foster rat mother model, whereby rat pups were breastfed during the entire lactation period, but received milk with a lower amount of Sia from PN3 until end of lactation; in addition, the animal intervention was performed at an early, developmental stage, which is challenging due to the vulnerability of pups; (2) the timeframe in which Sia content in brain and cognitive outcomes in rats were measured; previous analyses were carried out after weaning, when the Sia supplementation had just finished; herein, brain composition analyses and behavior assessments were performed at the end of Sia supplementation, but also in adult animals that had spent almost one year without receiving additional Sia; and (3) the use of 6′-SL as a source of Sia in comparison to the free form.

Improved cognitive skills reported here were not attributable to an increased Sia content in a brain hemisphere. In fact, Sia content in brain hemispheres samples was the same for the three experimental groups at weaning and in adulthood. According to Wang et al., the ganglioside and glycoprotein sialic acid concentrations in the brain frontal cortex are higher in breastfed infants when compared to formula-fed infants [22]. In a study performed with piglets, it was observed that supplementation with an exogenous source of Sia increased protein-bound sialic acid concentrations in the frontal cortex [28]. Jacobi et al. also demonstrated in piglets that a formula supplemented with dietary 6′-SL and 3′-SL increased the sialic acid bound to gangliosides in several areas of the brain [52]. As described above, Mudd and co-workers have recently published a study in which piglets were fed several diets containing different doses of sialyllactose from PN2–PN32 and showed that dietary sialyllactose increased conjugated Neu5Ac in the prefrontal cortex, among other brain structures [51]. In our study, Neu5Ac determination by HPLC was done in the whole hemisphere instead of in separate structures. Thus, significant differences among groups could have been missed because Neu5Ac may be accumulated in certain brain structures such as frontal cortex. Furthermore, it could be hypothesized that Neu5Ac content in the brain of the different groups was different during an early phase of development, promoting higher rates of axonal growth and enhanced connections between neurons that would support the behavioral differences found in adulthood.

To further explore this, we analyzed NCAM and PSA-NCAM expression levels in the frontal cortex of early weaned pups (PN22). NCAM is a widely-expressed protein involved in the stabilization and modulation of CNS [3]. Polysialic acid (PSA) is a linear homopolymer of α2-8-linked Neu5Ac. PSA is added to NCAM by a regulated post-translational process and varies through development [20]. Polysialylation supports the maintenance of an immature phenotype, allowing the neurites to grow and sprout to connect the complex circuitry of the brain while its graded downregulation enables fine-tuning of NCAM-dependent cell-cell interactions, stabilizing the newly-formed structures. Following the findings of the studies previously cited [28,52], our results in weaned rats showed that PSA-NCAM expression in the frontal cortex was significantly higher in the 6′-SL group when compared to Neu5Ac and the control groups; this result suggests that conjugated Sia such as 6′-SL might be taken up preferentially by the brain, enhancing the sialylation process of proteins highly involved in brain development. The maintenance of PSA-NCAM levels suggests neuroplasticity may be higher in the 6′-SL group.

With regards to cognitive evaluation, several behavioral tests were implemented consisting of two classical tests including the Y Maze with blocked arm and NORT, as well as a novel cognitive paradigm performed in the IntelliCage^®^ system. Y maze and NORT have been used in previous animal studies to evaluate the effects of nutrition on cognition [53,54] and were conducted at both ages in the present work. IntelliCage^®^ paradigm was only performed in adulthood. Data from weaned rats did not show differences among groups in any of the classical tests. In fact, performing cognitive tests at early ages of life entails certain methodological challenges. Leussis and Bolivar [55] pointed out that pre-weanling and weanling rodents normally habituate slower to novel environments, showing low exploratory activity when compared to older animals. It has been suggested that younger animals would perform better in rewarded tasks. Longer periods of exposure or habituation have also been proposed to increase the success of these tests. Based on our experience, the intense physical activity and the lack of focus in very young rodents are the main hurdles found when carrying out cognitive evaluation of weanling animals.

Conversely, significant differences were found in the assessment of cognitive skills in adulthood. In NORT, groups receiving Sia during lactation could distinguish better between novel and familiar objects than the control group. Impairment of the perirhinal cortex, the main brain structure involved in object recognition memory [44,56] has been suggested [57] as the reason why animals are not able to get a good score on memory tasks like NORT. Results obtained from the IntelliCage^®^ paradigm confirmed that animals receiving Sia at early stages performed significantly better than the control group in adulthood. As for the Y maze test, Sia-supplemented animals performed better than the control rats, since they were able to recognize the novel arm easily. An interesting finding to highlight was that, within groups supplemented with Sia, 6′-SL rats had better scores than animals that received Neu5Ac during lactation, suggesting 6‘-SL may be a better source of Sia.

An added value of the present study is that animal cognitive assessments were also supported by electrophysiological data based on in vivo LTP measurements. LTP is the basic mechanism for certain types of hippocampal learning [40]. The best results were observed in Sia-supplemented animals, mainly those of the 6′-SL group, while the control group performance was significantly lower than that of 6′-SL rats. These data suggest early supplementation with Sia might enhance processes related to neuronal plasticity.

Remarkably, control animals under-performed in all tests compared to Sia groups. In NORT, there were no differences in exploration time for both objects. In the Y maze, no preference for visiting the blocked arm first was shown. In the IntelliCage^®^, these animals scored poorly for correct visits, and a low increase in fEPSP amplitude was found in LTP. These results highlighted a certain degree of deficiency in control animals that could be explained by the lower amount of sialic acid and/or other nutrients such as minerals [58] that they received during the lactation period. The data indicate that the exogenous Sia provided by milk is necessary for the establishment of a neuronal network that ensures long-lasting cognitive skills. Lower levels of Sia during this critical timeframe may lead to premature aging of certain brain functions, while an exogenous supply of Sia was shown to be effective in maintaining cognitive skills for a longer duration. This phenomenon agrees with our previous research in which early supplementation during lactation with the HMO 2′-fucosyllactose (2′-FL) induced cognitive benefits in adult animals [42]. This new perspective highlights the concept of early nutrition [59] and the role of sialic acid in brain development.

Although the mechanism of action through which HMOs might exert this positive effect on cognitive outcomes is not yet known, there are several hypotheses. The improved cognitive performance observed in the Sia-supplemented groups could be due to an increase in the polysialylation of NCAM [60] or to an increase of sialylated compounds in brain due to the additional supply of Sia. There is a consistent body of evidence highlighting the potential biological importance of sialylated compounds in brain. Gangliosides are known to be key molecules in many CNS functions such as synaptogenesis, neurite and axonal growth and neural transmission [61,62,63].

Alternatively, 2′-FL orally given to adult mice also improves cognition and is dependent on gut-brain crosstalk through the vagus nerve [29]. The gut-brain axis (GBA) is a complex bidirectional network that communicates between the brain and gastrointestinal tract and modulates the respective functions [31]. This gut-brain crosstalk is driven by neural pathways and immune and endocrine mechanisms. It is known that one of the main factors able to trigger or modulate GBA is the intestinal microbiota [64] by producing different metabolites, such as short chain fatty acids or neurotransmitters. Several studies have reported how HMOs promote intestinal bacteria such as *Bifidobacterium* and *Lactobacillus* [65,66] based on their prebiotic role. Tarr et al. reported that dietary supplementation with both 6′-SL or 3′-SL effectively altered colonic microbiota communities, as well as diminished stressor-induced alterations in colonic mucosa structure, anxiety-like behavior and immature neuron cell numbers regardless of immune or endocrine functionality [30]. Despite this evidence, the role of 6′-SL as a GBA regulator and the mechanism underlying the reported effects are yet to be explored. Thus, the reported benefits on CNS function could be related either to its prebiotic capability, with its uptake by the brain during the critical neurodevelopment period, inducing long-lasting biochemical changes that ultimately enhance long-term cognitive skills or via GBA regulation.

With regards to the Sia sources used in this study, our results show that free Sia was less effective than 6′-SL, possibly because free Sia is more rapidly eliminated in the urine and, therefore, less bio-available. Nöhle and Schauer [67] suggested that metabolism of sialic acids bound to oligosaccharides might take more time than free Sia, since the former would require hydrolysis. In addition, oral treatment with 6′-SL in a mouse model of symptomatic *GNE* myopathy led to greater restoration of sialylation in muscle and improvement in muscle size and function as compared to the group treated with free Sia [68]. Based on these data, 6′-SL may be a better source of bio-available Sia.

## 5. Conclusions

In summary, our findings confirm the beneficial effects of supplementation of Neu5Ac or 6′-SL during lactation on long-term cognitive development in rats. Both compounds had positive outcomes; however, the results showed improved scores in behavior and electrophysiological analysis in those animals that received 6′-SL compared to Sia. Further research is needed to delineate the mechanisms of action underlying cognitive benefits and the potential role of intestinal microbiota.

## 6. Patents

There is one patent resulting from the work reported in this manuscript: the method of achieving memory and learning improvement by the administration of sialic acid. Publication number: WO/2015/085077. International Application Number: PCT/US2014/068582.

## Figures and Tables

**Table 1:** Concentration of Sia (µg Neu5Ac/mg) in brain in weaned and 1-year old rats supplemented with different Sia sources or water during the lactation period. Data are the mean ± SEM.

	Sia Concentration (µg Neu5Ac/mg Brain)			
	Neu5Ac Group	6′-SL Group	Control Group	One-Way ANOVA *p*-Value
Pups rats (*n* = 8/group)	1.399 ± 0.067	1.405 ± 0.124	1.422 ± 0.058	*p* = 0.8336
1-year old rats (*n* = 8–10/group)	1.018 ± 0.106	1.067 ± 0.025	0.9745 ± 0.096	*p* = 0.2143

Sia, sialic acids; Neu5Ac, N-acetylneuraminic acid; 6′-SL, 6′-sialyllactose.
